# Predicting Panel of Metabolism and Immune-Related Genes for the Prognosis of Human Ovarian Cancer

**DOI:** 10.3389/fcell.2021.690542

**Published:** 2021-07-12

**Authors:** Lingyun Zhang, Wenwen Sun, Weimin Ren, Jinguo Zhang, Guoxiong Xu

**Affiliations:** ^1^Department of Medical Oncology, Zhongshan Hospital, Fudan University, Shanghai, China; ^2^Cancer Center, Zhongshan Hospital, Fudan University, Shanghai, China; ^3^Department of Pathology, Shanghai First Maternity and Infant Hospital, Tongji University, Shanghai, China; ^4^Department of Pathology, Shanghai 9th People’s Hospital, Shanghai Jiao Tong University School of Medicine, Shanghai, China; ^5^Research Center for Clinical Medicine, Jinshan Hospital, Fudan University, Shanghai, China; ^6^Department of Oncology, Shanghai Medical College, Fudan University, Shanghai, China

**Keywords:** ovarian cancer, risk model, prognosis, metabolism, immune

## Abstract

**Objective:**

Ovarian cancer (OC) is a high deadly gynecologic cancer with a poor prognosis. The identification of genomic aberrations could predict the clinical prognosis of OC patients and may eventually develop new therapeutic strategies in the future. The purpose of this study is to create comprehensive co-expressed gene networks correlated with metabolism and the immune process of OC.

**Methods:**

The transcriptome profiles of TCGA OC datasets and GSE26193 datasets were analyzed. The mRNA expression level, hub genomic alteration, patient’s survival status, and tumor cell immune microenvironment of metabolism-related genes were analyzed from TCGA, GTEX, Oncomine, Kaplan-Meier Plotter, cBioPortal, TIMER, ESTIMATE, and CIBERSORT databases. We further validated the mRNA and protein expression levels of these hub genes in OC cell lines and tissues using qRT-PCR and immunohistochemistry.

**Results:**

The LASSO-Cox regression analyses unveiled seven differently expressed metabolism-related genes, including *GFPT2, DGKD, ACACB, ACSM3, IDO1, TPMT*, and *PGP*. The Cox regression risk model could be served as an independent marker to predict the overall clinical survival of OC patients. The expression of GFPT2, DGKD, ACACB, and ACSM3 were downregulated in OC tissues, while IDO1, TPMT, and PGP were upregulated in OC tissues than in control. Moreover, DGKD and IDO1 were significantly associated with the human immune system.

**Conclusion:**

The differently expressed metabolism-related genes were identified to be a risk model in the prediction of the prognosis of OC. The identified hub genes related to OC prognosis may play important roles in influencing both human metabolism and the immune system.

## Introduction

Human ovarian cancer (OC) is one of the most deadly gynecologic malignancies in the world (Siegel et al., 2020). Epithelial OC, the main type in histological, is regarded as originating from human epithelial cells of the ovary or the fallopian tube (Zhang et al., 2017). OC patients are usually diagnosed at the advanced clinic stage owing to the absence of symptoms at an early stage and the lack of an effective diagnostic marker. Although advances in surgery, chemotherapy, radiotherapy, immune and targeted therapy have been achieved great progress in OC treatment, the 5-year overall survival rate is still very low. One of the major reasons is the lack of effective markers to predict the prognosis which could give OC patients an early opportunity to change the treatment project. Thus, the exploration of new prognostic markers to distinguish and predict OC patients’ outcomes have a high clinical value.

Metabolic reprogramming is recently regarded as one of the new hallmarks of cancer (Hanahan and Weinberg, 2011). The previous work showed that one of the key enzymes of vitamin metabolism plays an important role in the process of OC and could be treated as a potential molecular biomarker of OC (Zhang et al., 2017). Recently, the dysfunction of oncogenes and tumor suppressors promotes metabolic reprogramming and enhances nutrient uptake to sustain energy supply and biomass synthesis (Boroughs and DeBerardinis, 2015). The difference in metabolism biology between tumor cells and normal cells makes drugs targeted metabolic pathways, which becomes a hot topic in cancer treatment. New agents focusing on metabolic genes were reported could eliminate tumor cells. The disorder of cell metabolisms such as accumulation of glycogen, fatty acid metabolism, and unsaturated lipids was found to take effect on the development of cancer (Iida et al., 2012). Even though the therapy targeted metabolism system in cancer treatment has proved to be effective, the molecular mechanism is poorly exploited (Khadirnaikar et al., 2020).

Besides, the other important hallmark of cancer is reported as immune infiltration (Hanahan and Weinberg, 2011). The human immune system plays a role in the beginning and process of tumors (Galon et al., 2013; Khadirnaikar et al., 2020). Since the metabolism and immune system are both the vital hallmarks of cancer, they may take part in the development of OC to a certain extent together. Immunotherapy which increases tumor-infiltrating T cells in tumors helps to clear tumor cells effectively because of the immune-active nature of tumors (Baert et al., 2017). The research showed that there was a significant correlation between high lymphocyte infiltration and the survival time in OC patients (Ovarian Tumor Tissue Analysis (OTTA) Consortium, Goode et al., 2017; Khadirnaikar et al., 2020). While the potential processes of the human immune system in some cancer types have been clarified, the mechanism of immune-related genes in OC is still not very clear. And to our knowledge, until now there is no research focusing on the genes of OC from both the metabolism system and immune system simultaneously.

The study was aimed to identify the differently expressed metabolism-related genes and try to further declare their association with the human immune system. We analyzed the correlation between the expression levels of these genes with clinical characteristics. The underlying molecular regulatory mechanisms were also further elucidated through bioinformatics analyses.

## Materials and Methods

### Public Data Mining

The Cancer Genome Atlas database (TCGA^1^) and Genotype-Tissue Expression database (GTEX^2^), which contains 379 OC surgical specimens and 88 normal ovarian tissue for conducting the primary analysis. The transcriptome data of GSE26193 containing 107 OC samples with clinical characteristics (Mateescu et al., 2011) were further retrieved from the Gene Expression Omnibus database (GEO^3^). Patients who received chemotherapy, radiotherapy and treatment before surgery were excluded. Probes were included only with gene annotation and matched with one gene symbol.

### Detection of Differently Metabolism-Related Genes

| logFC| > 1 and *P*-value < 0.05 was set as the cut-off criteria. We screened 91 differently expressed metabolism-related genes by the “limma” R package in TCGA-OV dataset compare to GTEX normal tissues. The mRNA expression was validated by the Gene Expression Profiling Interactive Analysis database (GEPIA^4^) and the Oncomine database^5^ (Wen et al., 2020).

### Functional Enrichment Analysis and PPI Network Construction

Gene ontology (GO) (Ashburner et al., 2000) and Kyoto Encyclopedia of Genes and Genomes (KEGG) pathway enrichment analyses (Kanehisa et al., 2017) were used to investigate the biological functions of the differently expressed metabolism-related genes by the R package (Ritchie et al., 2015). The Protein-Protein Interaction (PPI) network with high confidence was established from the Search Tool for the Retrieval of Interacting Genes (STRING) database^6^ (Szklarczyk et al., 2019).

### Establishment of Prognostic Risk Model

TCGA-OV dataset was treated as a training cohort. GSE26193 dataset was adjusted as the external validation cohort. The expression data of the differently metabolism-related genes from the TCGA-OV dataset were employed to establish a risk score model. The impact of each differently expressed metabolism-related gene on OS of OC patients was further to be valued via the univariate Cox proportional risk regression model. Log-rank *P* < 0.05 was considered statistically significant. LASSO-Cox regression was used to minus the scope of genes (Tibshirani, 1997). Internal and external validation cohorts were then used to verify the robustness of the risk model. Moreover, the clinical indexes and risk scores were included in univariate and multivariate Cox regression analyses to validate the independence of the risk model. A cutoff value by the Gordon index was used to divide the high-risk and low-risk groups (Wen et al., 2020). A log-rank test was used in comparing the survival difference between the two groups. The OS of each group was performed using the Kaplan–Meier (KM) survival curve.

### Survival Analysis of Metabolism-Related Genes in Kaplan-Meier Plotter

The OS, PFS, and clinical stage of OC patients were performed from the Kaplan–Meier Plotter^7^. The 95% confidence intervals (CIs) of hazard ratio (HR) and log-rank *P*-value were counted.

### Oncomine Analysis

The mRNA levels of the seven differently expressed metabolism-related genes in OC and normal control tissues were analyzed from the Oncomine database^8^. Fold-change = 1.5, *P* = 0.05, mRNA data type were determined as the thresholds.

### cBioPortal Database Analysis

The cBioPortal^9^ was applied for estimating the genetic alterations of the differently expressed metabolism-related genes. The mRNA expression z scores (RNA Seq V2 RSEM) were got with a z score threshold of ± 2.0 (Zhang et al., 2020). An overview of genetic alterations is presented by OncoPrint.

### Correlation of Differently Metabolism-Related Genes With Immune Cells

Further research of the correlation of seven differently expressed metabolism-related genes with immune cells was analyzed in The Tumor IMmune Estimation Resource (TIMER^10^). ESTIMATE (Estimation of STromal and Immune cells in MAlignant Tumors using Expression data) was used to measure the fraction of immune cells in tumor samples using gene expression profile. CIBERSORT (Cell-type Identification By Estimating Relative Subsets Of RNA Transcripts) is a deconvolution algorithm that characterizes the cell composition of complex tissues from gene expression profiles. In the present study, TCGA OC samples were analyzed via the CIBERSORT algorithm to describe 22 immune cells by *P* < 0.05. The violin plot of associations between DGKD, IDO1 expression, and 22 immune cells was produced with “vioplot” and “limma” packages in R 4.02 (Zhang et al., 2021). Scatter plots of the correlation of tumor-infiltrating immune cells proportion with the PNPO expression were plotted with “ggplot2,” “ggpubr,” and “gridExtra” R packages.

### qRT-PCR

Total RNA was collected and extracted from human non-tumorous human ovarian surface epithelial cells (HOSEpiC), human OC cell lines SK-OV-3 (adenocarcinoma), OVCAR-3 (adenocarcinoma), ES-2 (clear cell carcinoma), and A2780 (human ovarian carcinoma) by the RNA-Quick Purification Kit (Yishan Biotechnology Co., Ltd., Shanghai, China). The primer sequences were shown in Supplementary Table 1.

### Immunohistochemistry

The study of the human subject was approved by the Ethics Committee of Shanghai First Maternity and Infant Hospital, Tongji University. Human OC tissues and control tissues were got from Shanghai First Maternity and Infant Hospital. Five ovarian tissue samples were obtained from patients with OC. Five control ovarian tissue samples and five control fallopian tube tissue samples were obtained from patients with non-tumorous ovaries and fallopian tubes, respectively. The following antibodies anti-ACSM3 (#10168-2-AP), anti-TPMT (#10682-1-AP), anti-IDO1 (#13268-1-AP), anti-ACACA (#21923-1-AP), and anti-PGP (#25081-1-AP) were obtained from ProteinTech Group (Chicago, IL, United States). Anti-DGKD (#abs111548) and anti-GFPT2 (#abs111910) were obtained from Absin Bioscience Inc. (Shanghai, China).

### Statistical Analysis

Statistical analyses of bioinformatics results were used by R software version 3.6.1^11^ and performed by GraphPad Prism 8.0 (GraphPad Software Inc., San Diego, CA, United States). A log-rank test was used to compare the high-risk with the low-risk group in the Kaplan-Meier curve. Different expression of seven hub genes between two groups of experimental results was compared by a two-tailed Student’s *t*-test. Data were shown as mean ± the standard deviation. *P* < 0.05 was considered as statistical significance.

## Results

### Identification of Differently Expressed Genes in OC

The flowchart of the analysis process is displayed in Supplementary Figure 1. The gene expression profiles were got from the dataset TCGA and GTEX database, which contains 379 OC surgical specimens and 88 normal ovarian specimens. The heat map of differently expressed genes between human OC tissue and ovarian normal tissue was shown in Supplementary Figure 2. Function annotation analyses were then executed. GO enrichment suggested that the differently expressed genes were major consisted of extracellular structure organization, extracellular matrix, and cell adhesion molecular binding (Supplementary Figure 3A). KEGG enrichment indicated that the hub genes were mainly related to the cell cycle, Ras signaling pathway, and Platinum drug resistance (Supplementary Figure 3B).

### Identification and Bioinformatics of Hub Genes in OC

The heatmap of total differently expressed metabolism-related genes between OC surgical tissues and control normal ovarian tissues were extracted (Supplementary Figure 4A). The PPI network of the differently expressed metabolism-related genes was constructed by the STRING database (Supplementary Figure 4B). GO analysis enlightened that the differently metabolism-related genes were mainly related to small molecular catabolic processes, mitochondrial matrix, and coenzyme binding (Supplementary Figure 4C). KEGG analysis indicated that the genes were mainly associated with drug metabolism, glycerophospholipid metabolism, and glutathione metabolism (Supplementary Figure 4D).

### Validation of the Prognostic Risk Model

**FIGURE 1:**
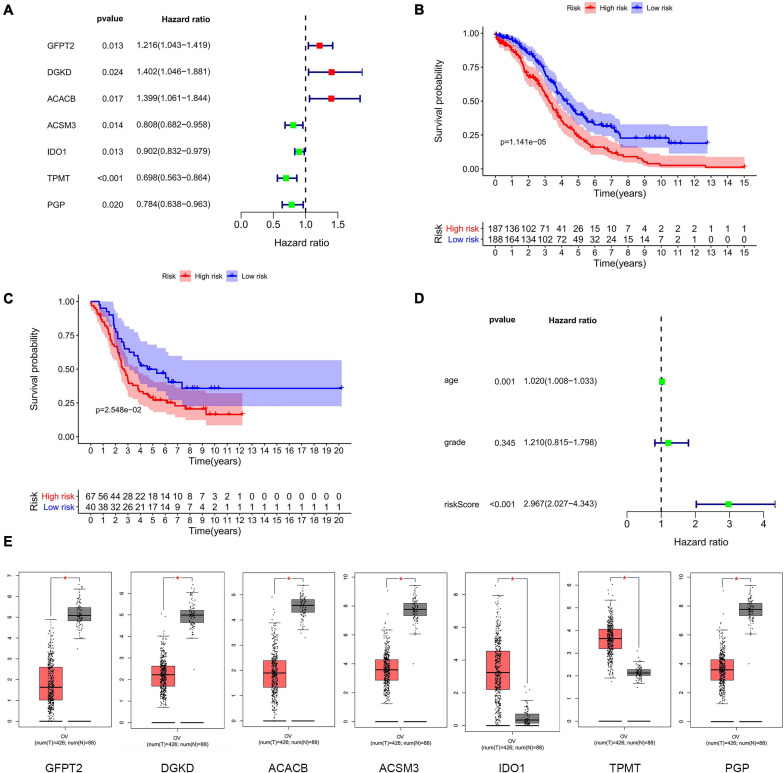
The differently expressed metabolism-related genes panel. **(A)** Establishment of the seven metabolism-related genes’ panel correlated to OC patients outcomes. Red, high-risk genes; Green, low-risk genes. **(B)** Kaplan-Meier curve analysis of seven metabolism-related genes with OS in the training dataset of TCGA. Red, high-risk group; Blue, low-risk group. **(C)** Kaplan-Meier curve analysis of seven metabolism-related genes with OS in validation dataset of GEO. Red, high-risk group; Blue, low-risk group. **(D)** Forrest plots in the training cohort. **(E)** The transcription level of seven metabolism-related genes between human OC and normal tissues in TCGA. Red, tumor group; Gray, normal group.

To determine the correlation with the prognosis, seven differently expressed metabolism-related genes (four low-risk genes and three high-risk genes) were screened as hub genes to be analyzed with overall survival (OS) of OC patients in TCGA (Figure 1A). OC patients were separated into a high-risk group (*n* = 187) and a low-risk group (*n* = 188) by the median risk score in the training cohort (TCGA dataset). We identified the prognostic outcome was worse in the high-risk group than the low-risk group (*P* < 0.01) (Figure 1B). The results of the verification cohort (GSE26193) were similar to the training group (Figure 1C). The risk score has a significant correlation with the OS by univariate (Figure 1D) and multivariate (Supplementary Figure 5) Cox regression analysis. Thus, the results suggested that the risk score was a better factor to predict the prognosis of OC than other clinical indices, such as age and stage. The mRNA expression level of each differently metabolism-related gene was extracted from the GEPIA database. The mRNA expression of GFPT2, DGKD, ACACB, and ACSM3 were downregulated in OC patients, while IDO1, TPMT, and PGP were upregulated in OC patients (Figure 1E). It was shown that most genes in the risk model had different expressions in OC mRNA level compared with normal ovarian tissues (*P* < 0.05).

### Prognostic Value of the Risk Model

**FIGURE 2:**
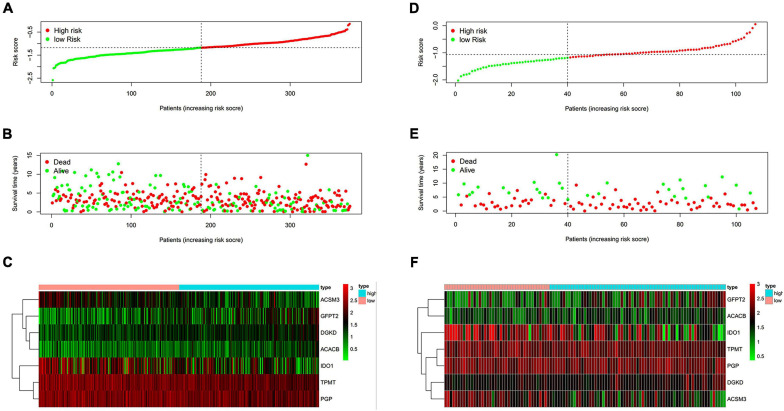
Evaluation of the performance of the risk model. **(A,D)** Distribution of risk score in patients. **(B,E)** Survival status scatter plots of patients (red dots represent death, blue dots represent alive). **(C,F)** The seven-gene expression patterns in the training cohort. **(A–C)** from TCGA database. **(D–F)** from GEO database.

The risk score of patients was ranked (Figure 2A) and the survival state of patients was shown by dot chart (Figure 2B) in the training cohort. The hub genes were separated into a high-risk and low-risk group which were shown in the heat map (Figure 2C). It was suggested that patients in a high-risk score group had worse OS than in a low-risk group. Similarly, the risk score, dot chart, and heat map were similar to the training group (Figures 2D–F). The results concluded that the risk model could be served as a prognostic factor of OC patients.

### Analysis of Hub Genes and Clinical Data

**FIGURE 3:**
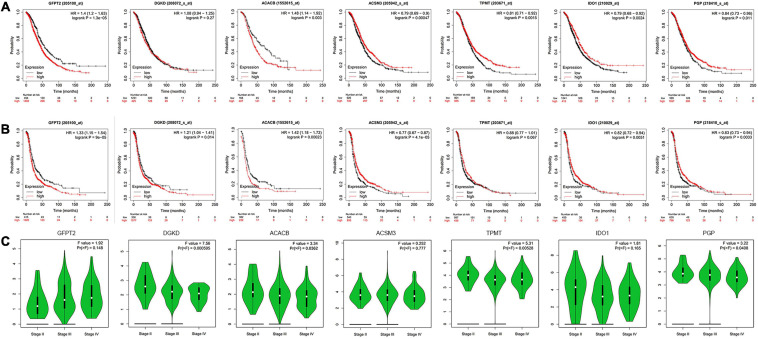
Analysis of hub genes with clinical data. **(A)** Kaplan-Meier curve analysis of OS comparing the high and low levels of seven metabolism-related genes. **(B)** Kaplan-Meier curve analysis of PFS comparing the high and low expression levels of seven metabolism-related genes. **(C)** The violin pot shows the correlations with the clinical stage. Red, high expression; Blue, low expression.

To investigate the association between metabolism and OC patients’ clinical data, we analyze the seven metabolism-related genes with OS, progression-free survival (PFS), and clinical stage. The analysis results indicated that the expression of GFPT2, ACACB, ACSM3, IDO1, TPMT, and PGP had a remarkable association with patient OS (Figure 3A), while the expression of GFPT2, DGKD, ACACB, ACSM3, IDO1, and PGP were remarkably associated with patient PFS (Figure 3B). For OS analysis, higher expression of GFPT2 and ACACB indicated poorer OS, while higher expression of ACSM3, IDO1, TPMT, and PGP indicated better patient OS (*P* < 0.05). For PFS analysis, higher expression of GFPT2, DGKD, and ACACB indicated poorer PFS, while higher expression of ACSM3, IDO1, and PGP indicated better patient PFS (*P* < 0.05). Exploration of the differently metabolism-related genes during the clinic progression of OC showed that the levels of DGKD, ACACB, TPMT, and PGP were significant correlated with the clinical stage (Figure 3C). Thus, these results showed that these genes contribute to the evolution of OC (*P* < 0.05).

### Genetic Alterations in OC Patients

ACSM3 and IDO1 were significantly overexpressed in OC, while GFPT2, DGKD, and ACACB were significantly decreased in the Oncomine database (Supplementary Figure 6A). The relevant mRNA expression data of TPMT and PGP in OC were absent in the Oncomine database, but they were found to be upregulated in OC in the TCGA database. The analysis of genetic alterations of the hub genes was analyzed from cBioPortal. Amplification, mRNA high, deep deletion, and mutation were found in the high-risk group, while amplification, mRNA high, and deep deletion appeared in the low-risk group. TPMT had the most frequent genetic alterations (15%), while amplification was its most popular genetic alteration (Supplementary Figures 6B, 7).

### Expression of the Hub Genes in Human OC Cell Lines and Tissues

**FIGURE 4:**
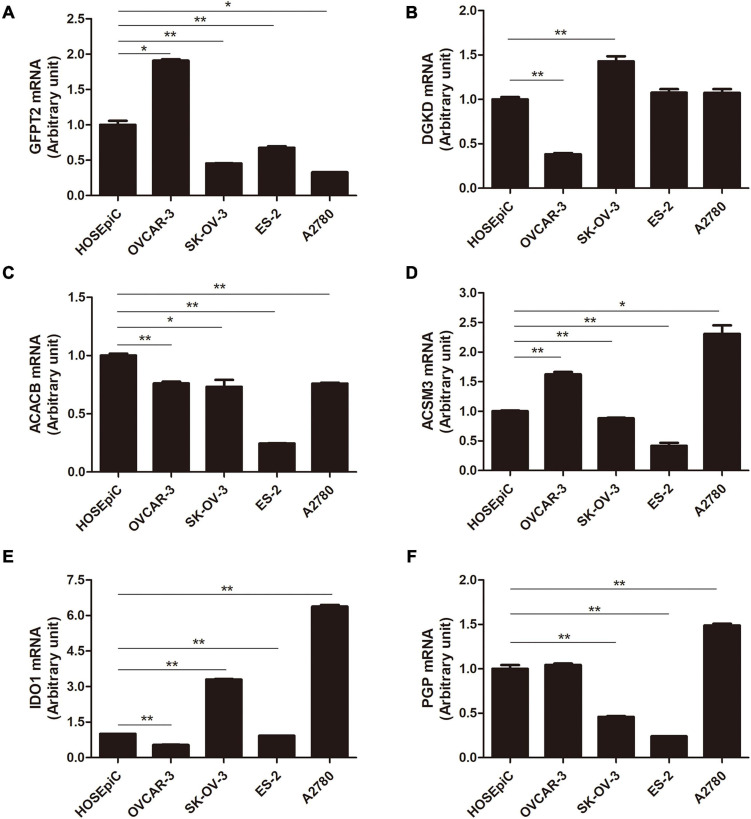
The mRNA level of six hub genes in normal ovarian cell line and OC cell lines by qRT-PCR. **(A)** GFPT2, **(B)** DGKD, **(C)** ACACB, **(D)** ACSM3, **(E)** IDO1, and **(F)** PGP. *P < 0.05, **P < 0.01.

**FIGURE 5:**
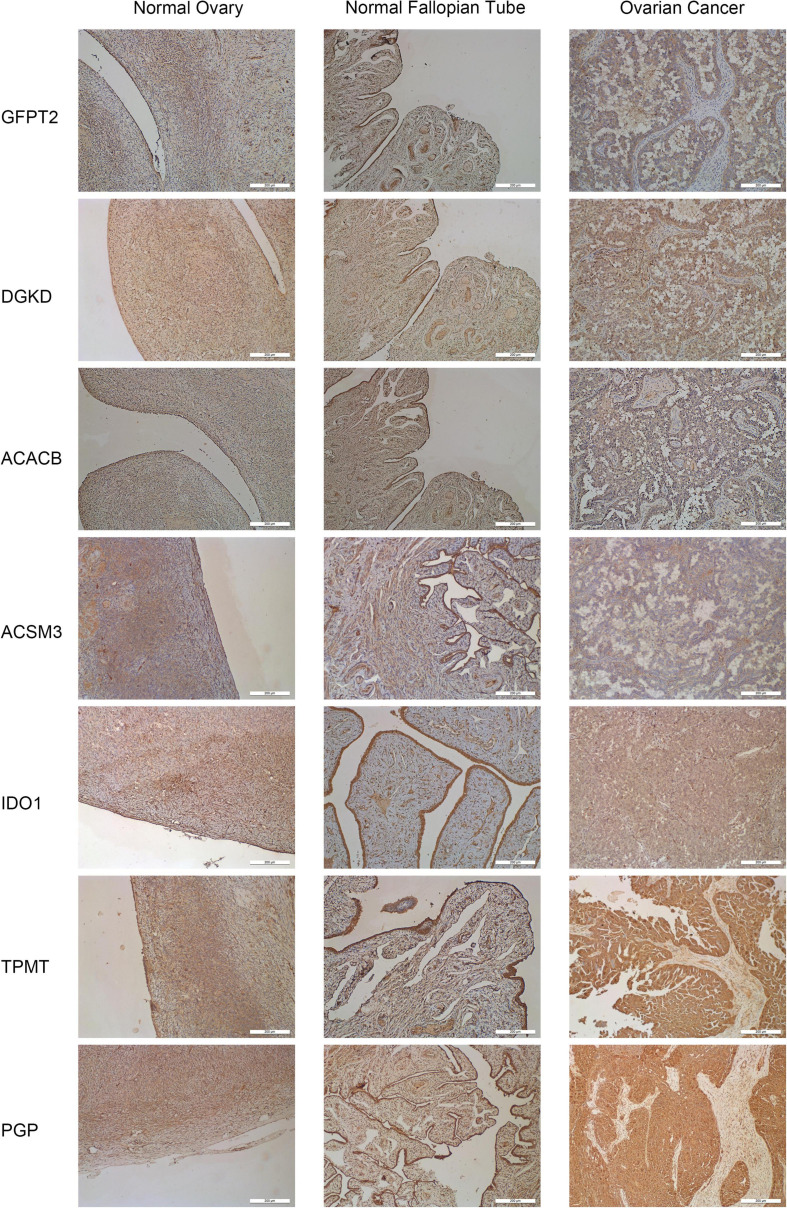
Representative immunohistochemistry staining for seven hub genes in normal ovarian tissues, normal fallopian tube tissues, and OC tissues. The staining of IDO1, TPMT, and PGP was strong in OC tissues. Magnification × 200.

To further assess the seven metabolism-related gene expression levels in OC, we perform qRT-PCR to search the mRNA expression of the hub genes in human OC cell lines (Figure 4). The mRNA expression of GFPT2 was decreased in SK-OV-3, ES-2, and A2780 cell lines than in the control non-tumorous HOSEpiC cell line. DGKD mRNA expression was decreased in OVCAR-3 than HOSEpiC cell line. ACACB mRNA level was lower expressed in all OC cell lines we tested. ACSM3 mRNA expression was decreased in SK-OV-3, OVCAR-3, ES-2, A2780 cell lines than the control cell line. The mRNA expression of IDO1 was higher in SK-OV-3, A2780 than in the HOSEpiC cell line, while PGP mRNA expression was higher in A2780 cells than in control. We also tested the mRNA expression of TPMT, but there was no significant value so we did not show it here. Interestingly, we found the hub genes expression level maybe not very consistent among different cell lines, but the general expression level trend was the same as the results that we extracted from the online database. The protein expression levels of GFPT2, DGKD, ACACB, and ACSM3 were lower in OC tissues than normal ovarian and fallopian tube tissues. While the protein levels of IDO1, TPMT, and PGP were overexpressed in OC tissues than in control (Figure 5).

### Correlation With Immune Cell Infiltration in OC Patients

**FIGURE 6:**
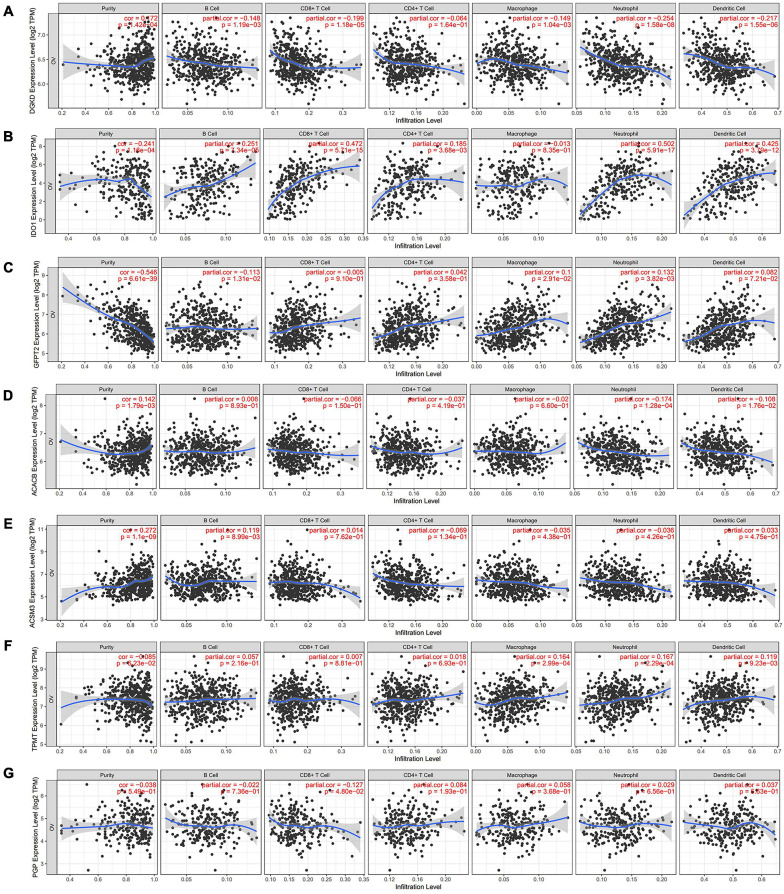
Analysis of immune cells infiltration with seven hub genes expression in TIMER database. The correlation between the infiltration of immune cells and the expression of DGKD **(A)**, IDO1 **(B)**, GFPT2 **(C)**, ACACB **(D)**, ACSM3 **(E)**, TPMT **(F)**, and PGP **(G)**.

Metabolism and immune disorder were both the important hallmarks of cancer, and they could cross-talk in some content during the process of cancer. We further investigate the correlation in the TIMER database. Two of the hub genes, DGKD and IDO1 have the most significantly association with the infiltration of immune cells. Obviously, a negative correlation between DGKD and the infiltration of B cells (Cor = −0.148, *P* = 1.19e–03), CD8 + T cells (Cor = −0.199, *P* = 1.18e–05), macrophages (Cor = −0.149, *P* = 1.04e–03), neutrophils (Cor = −0.254, *P* = 1.58e–08), and dendritic cells (Cor = −0.217, *P* = 1.55e–06) were found, when DGKD was positively associated with the purity (Cor = 0.172, *P* = 1.42e–04; Figure 6A). On the contrary, a positive interrelation between IDO1 expression and the infiltration of B cells (Cor = 0.251, *P* = 7.34e–05), CD8 + T cells (Cor = 0.472, *P* = 5.71e–15), CD4 + T cells (Cor = 0.185, *P* = 3.68e–03), neutrophils (Cor = 0.502, *P* = 5.91e–17), and dendritic cells (Cor = 0.425, *P* = 3.79e–12) were showed, while IDO1 was negatively related with the purity (Cor = −0.241, *P* = 1.16e–04; Figure 6B). Moreover, we also found GFPT2 expression was positively associated with macrophages and neutrophils (Figure 6C), while ACACB expression was negatively correlated with neutrophils and dendritic cells (Figure 6D). However, there is no significant correlation of the expression of ACSM3, TPMT, and PGP with infiltrating levels of immune cells infiltration (Figures 6E,F,G).

**FIGURE 7:**
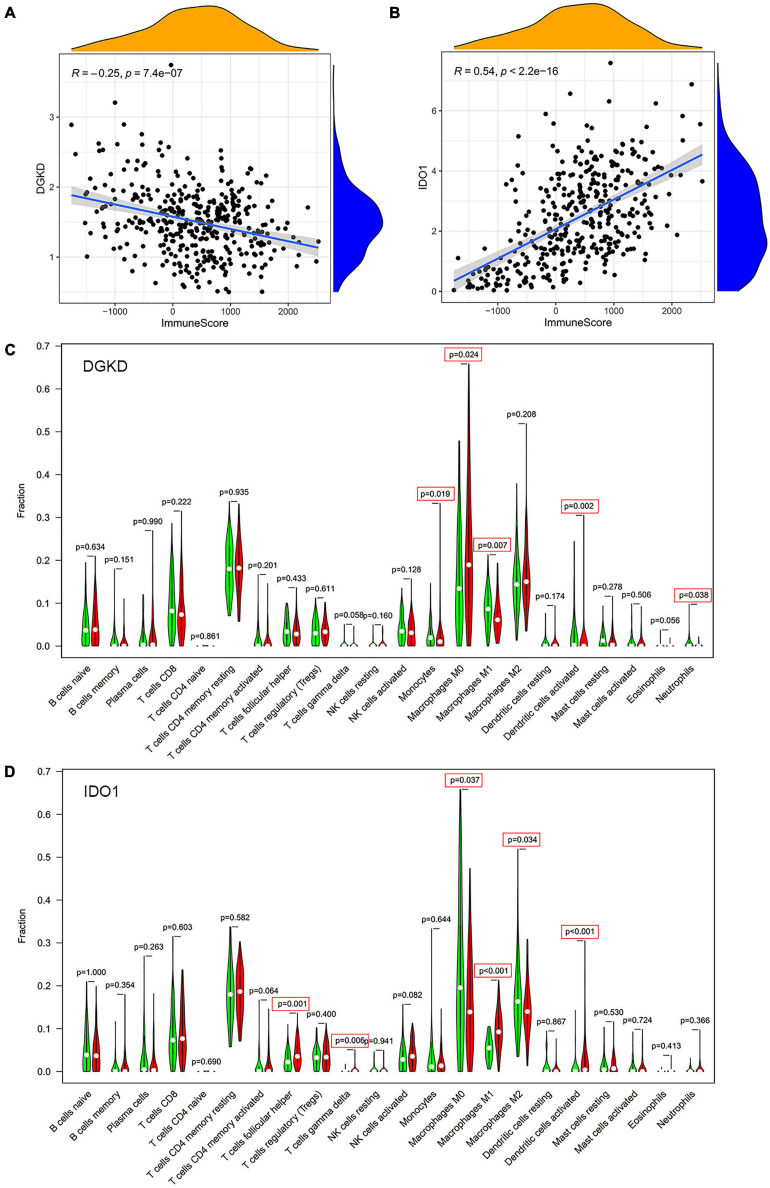
Analysis of immune cells infiltration with DGKD and IDO1 expression in CIBERSORT database. The negative correlation of DGKD expression with immune cells **(A)** and the positive correlation of IDO1 expression with immune cells **(B)**. The Violin plot of immune cells between OC tumor samples with the low or high expression level of DGKD **(C)** and IDO1 **(D)** relative to its median expression level, respectively. Red, high expression; Green, low expression.

To further exam the relationship of DGKD and IDO1 with the organic immune system, tumor-infiltrating immune cells were searched through CIBERSORT and ESTIMATE. It showed a negative relationship between DGKD and immune cells infiltration (*R* = −0.25, *P* = 7.4e–07, Figure 7A), while a positive association was found between IDO1 expression and immune cells infiltration (*R* = 0.54, *P* < 2.2e–15, Figure 7B) in CIBERSORT database. The data showed that five types of tumor-infiltrating immune cells were significantly correlated with DGKD expression, including macrophages M0, monocytes, and dendritic cells activated were positive with DGKD, while macrophages M1 and neutrophils were negative with DGKD expression (*P* < 0.05, Figure 7C). And six kinds of tumor-infiltrating immune cells have a significant correlation with IDO1, including T cells gamma delta, macrophage M1, T cells follicular helper, and dendritic cells activated were positive with IDO1 expression, while macrophages M0 and macrophages M2 were negative with IDO1 expression (*P* < 0.05, Figure 7D). Similarly, we found the infiltration of B cells memory, T cell follicular helper, T cells gamma delta, dendritic cells activated, macrophages M1, NK cell activated, and eosinophils were negatively correlated with the expression of DGKD while macrophages M0 and NK cells resisting were positively correlated with the expression of DGKD in ESTIMATE database (Supplementary Figure 8A). The T cells gamma delta, T cells follicular helper, T cells CD4 memory activated, dendritic cells activated, and macrophages M1 were positively related with IDO1 while macrophages M0 and macrophages M2 were negatively associated with IDO1 in the ESTIMATE database (Supplementary Figure 8B). These results further proved the expression levels of DGKD and IDO1 could affect the immune system of OC patients.

### Correlation of PNPO With Hub Genes in OC Patients

**FIGURE 8:**
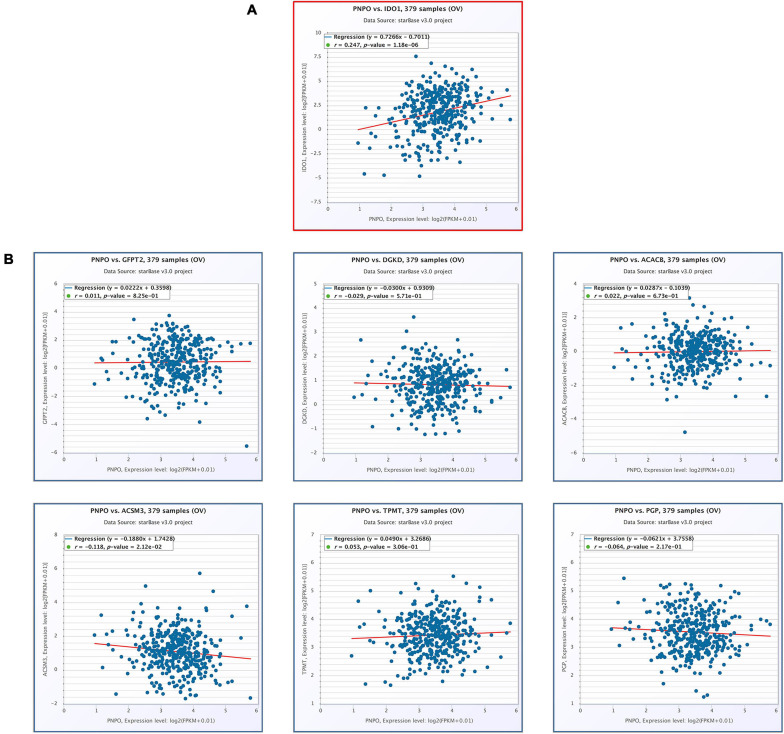
The correlation of seven hub genes with the expression of PNPO in the TCGA-OC cohort (starBase). **(A)** The positive correlation between PNPO and IDO1. **(B)** The correlation between the expression of PNPO and the other six hub genes.

We found PNPO took an essential effect on the progress of OC from our previous work. PNPO was one key enzyme of vitamin B6 and participated in human many physical metabolism processes. The present study showed that the seven hub genes were anticipating the metabolism process of human OC and were associated with immune cell infiltration. We deduced PNPO may have crosstalk with these genes from metabolism and immune process. Then we examined the correlation of PNPO with seven hub genes via TCGA ovarian cancer datasets. It showed a positive correlation between PNPO and IDO1expression (Figure 8A), while there was no correlation between the expression of PNPO and the other six hub genes (Figure 8B). We further found the infiltration of NK cells resisting, macrophages M1 and eosinophils were positively related with the expression of PNPO in the ESTIMATE database (Supplementary Figure 9A), although there was no significant correlation in the TIMER database (Supplementary Figure 9B). The data indicate that PNPO may take part in the regulation of metabolism and the immune system in OC patients.

## Discussion

Many Human OC patients were usually diagnosed in an advanced stage in the world. The OC patients generally have a good response to therapy but in turn to recurrence very soon. Until now, there is no excellent molecular biomarker to assess the prognosis of OC. Therefore, it is urgent to explore novel biomarkers to accurately assess OC patient outcomes. As we knew, cancer is a comprehensive process that depending on the interaction among cancer cells, the microenvironment, and the physical immune system. Increasing evidence revealed that the dysfunction of metabolism and immune response could affect the development of cancer.

The current study used TCGA to analyze the mRNA transcriptome of OC patients. We identified the differently expressed metabolism-related genes from a training cohort and perform a firm risk model to predict the survival rate of OC patients. We found a panel of different metabolism-related genes, concluding GFPT2, DGKD, ACACB, ACSM3, IDO1, TPMT, and PGP were correlated with OC patients’ survival. Furthermore, comprehensive analyses of the seven differently metabolism-related genes at mRNA and protein levels, genetic alterations were carried out. Besides, we further investigate the correlation of the candidate hub genes with the body immune microenvironment from the TIMER, CIBERSORT, and ESTIMATE databases. At last, the genes both associated with human metabolism and immune system were screened.

From our previous work, we found pyridoxine 5′-phosphate oxidase (PNPO) is overexpressed in human OC tissues and could be served as a potential therapeutic target (Zhang et al., 2017). PNPO works as one key enzyme in vitamin B6 metabolism, converting pyridoxine 5′-phosphate and pyridoxamine 5′-phosphate into pyridoxal 5′-phosphate, which serves as a co-factor for more than 150 enzymes constituting about 4% of human enzyme activities (Ueland et al., 2017). Although we did not find PNPO as one of the candidate hub genes of the penal predicting OC patients of prognosis from an online network, we found PNPO plays an essential role in the human metabolism process. The results of PNPO had a positive correlation with IDO1 suggesting that the interaction of different metabolism processes may exist on the development of OC. We further found PNPO has a positive relationship with the infiltration of NK cells resisting, macrophages M1, and eosinophils. These data indicated that PNPO took part in the development of OC not only by affecting the organic metabolism but also by influencing the human physical immune system.

IDO1 is a rate-limiting oxidoreductase that catalyzes tryptophan to kynurenine (Takikawa, 2005). Tryptophan is an essential amino acid needed by the physical metabolism to construct proteins for cellular growth and immune function. IDO1 might restrict the inflow of tryptophan from blood to tumor and produce tumor-toxic metabolites in endothelial cells, thus decreasing tumor growth (Riesenberg et al., 2007; Feng et al., 2020). We found IDO1 was a low-risk gene and the overexpression of IDO1 may predict a good prognosis for OC patients. It is suggested that the higher expression level of IDO1 has a positive correlation with OS and PFS in OC, which was consistent with our results (Feng et al., 2020). A positive correlation was found between IDO1 and dendritic cells, macrophages, and T cells infiltration (Li et al., 2018; Feng et al., 2020). It was shown that IDO1 is an essential factor in the innate immune system and could be induced by IFN-γ which is mainly produced by activated infiltrating CD8 + and CD4 + T cells (Takikawa et al., 1988; Feng et al., 2020). However, IDO1 was also reported to be related to poor prognosis in some cancer and used as a checkpoint by tumors to escape immune surveillance (Ino, 2011; Tsai et al., 2019; Zhang et al., 2019; Steeneck et al., 2020). It was viewed as a mediator of the immune system in some cancer (Cesario et al., 2011; Kristeleit et al., 2017). The expression of IDO1 seems to decrease tumor infiltration of B cells and to increase the infiltration of regulatory T lymphocytes (Carvajal-Hausdorf et al., 2017). The complexity of this phenomenon may be partly because IDO1 is expressed in two compartments, including tumor cells and tumor-infiltrating lymphocytes. The tumor microenvironment could be influenced by their interaction, result in variant outcomes of tumor prognosis (Feng et al., 2020). These data suggesting us physical energy metabolism genes may take effect by regulating the immune environment in the development of OC. Our results also showed that IDO1 and PNPO have a positive correlation, indicating both tryptophan and vitamin metabolism were involved in the development of OC. How the way of the crosstalk between these two metabolic pathways needs further research.

DGKD, a lipid kinase that terminates diacylglycerol signaling, catalyzes diacylglycerol phosphorylation to produce phosphatidic acid (Kanoh et al., 1990; Murakami et al., 2020). Diacylglycerol was important in lipid metabolism (Chibalin et al., 2008). Intracellular diacylglycerol and phosphatidic acid levels are usually under dynamic equilibrium, and disturbances of this balance may lead to the disorder of cellular metabolism (Chibalin et al., 2008). DGKD is one key enzyme involved in cellular signal transduction (Imai et al., 2002). It was discovered that DGKD overexpression could protect and improve glucose homeostasis in diabetes by AMPK-related signal transduction and lipid metabolism (Jiang et al., 2016; Wada et al., 2016). DGKD regulated the de-ubiquitination of EGFR through targeting Akt and USP8 or by modulating PKC signaling (Crotty et al., 2006; Cai et al., 2010) and decreased the expression of cyclin D1 (Sakai et al., 2018). We found DGKD was a high-risk gene that predicting a poor prognosis of OC patients. We also found DGKD was low expressed in OC patients, and DGKD has a negative correlation with B cells, CD8 + T cells, macrophages, neutrophils, dendritic cells, and NK cells infiltration. To our knowledge, there was no relative report of DGKD in human OC until we prepare this article. The data indicated that DGKD could act as a candidate prognosis indicator gene in OC by affecting physical metabolism and the immune system.

GFPT2 was a key enzyme of the hexosamine biosynthesis pathway that was investigated to anticipate the biology of colorectal cancer cells via enhancing the glycosylation of p65 and activating the NF-κB pathway (Zhang et al., 2018). GFPT2 was discovered to be overexpressed in mesenchymal cell lines (Simpson et al., 2012). The data showed that GFPT2 had a positive association with the clinical stage, which was overexpressed in OC tissues. The down-regulated expression of GFPT2 in OC cells could reduce β-catenin nuclear location and the expression level of Slug and Zeb1 (Zhou et al., 2019). Our data also suggest the GFPT2 was a high-risk gene in OC and is related to poor prognosis in OC.

ACACB is one isoform of acetyl-CoA carboxylase which controls fatty acid β-oxidation (McGarry et al., 1978; Hunkeler et al., 2018). The inhibition of fatty acid oxidation through ACACB could perturb tumor cells absorbing energy (Gonzalez-Angulo et al., 2012; Lu et al., 2019). ACACB has studied metabolic syndrome, obesity, colorectal cancer, diabetes diseases, lung cancer, and hepatocellular carcinoma (Svensson et al., 2016; Yu et al., 2018; Lally et al., 2019). Meanwhile, ACACB was served as a prognostic indicator in breast cancer patients receiving neoadjuvant chemotherapy (Klintman et al., 2016; Lu et al., 2019). ACACB is overexpressed in liver cancer and is highly associated with the prognosis (Ye et al., 2019). We found ACACB was a high-risk gene in OC and is related to poor survival.

The expression of ACSM3 was decreased in hepatocellular carcinoma tissues. And ACSM3 was found to be correlated with poor survival in advanced hepatocellular carcinoma (Gopal et al., 2017; Ruan et al., 2017). Loss of ACSM3 expression was suggested with the activation of the TGFβ, MYC, and WNT signaling pathways (Ruan et al., 2017). There was a positive association between ACSM3 and CD8 + T cells, macrophages, T regulatory cells, and dendritic cells in melanoma (Zhu et al., 2020). In our present study, ACSM3 was decreased in OC and the downregulation of ACSM3 was found to be correlated with poor survival time of OC patients. But there was no correlation between ACSM3 and the immune microenvironment in OC.

TPMT is an enzyme that took an important effect in the metabolism of immunosuppressant azathioprine and mercaptopurine drugs mainly in rheumatoid arthritis, pediatric leukemia, and inflammatory bowel disease (Booth et al., 2011). Several studies have shown that the absence of TPMT activity may be associated with increased risk for drug-related toxicity of patients, including myelosuppression, hepatotoxicity, and pancreatitis (Meggitt and Reynolds, 2001; Gisbert and Gomollon, 2008). TPMT deficiency is associated with severe bone marrow toxicity and the heterozygote of TPMT is an increased risk factor of myelosuppression (Lennard, 2014). PGP has been described as a phosphatase. PGP targets phosphoglycolate, which resulted largely from a side activity of pyruvate kinase (Segerer et al., 2016). PGP was a metabolic repair enzyme that could regulate central carbon metabolism (Linster et al., 2013; Dumont et al., 2019). Until now there is no research on TPMT and PGP of OC. We found that TPMT and PGP were overexpressed in OC patients and had a significant correlation with OS and clinical stage. Combined with the other five genes, the seven genes panel could be the candidate prognosis biomarkers of OC.

## Conclusion

We used the seven hub genes panel as a molecular signature to build a risk model which could accurately predict the prognosis in OC patients. High-risk and low-risk of OC patients can be screened by this panel that may help oncologists to provide a more proper therapeutic strategy for patients with OC. Besides, *DGKD* and *IDO1*, two genes of the panel, were significantly correlated with the physical immune system, suggesting that metabolism and immune microenvironment may play crosstalk efforts in the development of OC.

## Data Availability Statement

The datasets presented in this study can be found in online repositories. The names of the repository/repositories and accession number(s) can be found in the article/Supplementary Material.

## Ethics Statement

The studies involving human participants were reviewed and approved by the Ethics Committee of Shanghai First Maternity and Infant Hospital, Tongji University (Ethical committee No. KS21161). The patients/participants provided their written informed consent to participate in this study. Written informed consent was obtained from the individual(s) for the publication of any potentially identifiable images or data included in this article.

## Author Contributions

LZ and WR developed the idea and designed the research. LZ, WS, and JZ analyzed the data. LZ and JZ performed cell culture and PCR. WS performed the IHC. LZ, WS, and WR wrote the draft of the manuscript. GX supervised the study and edited the manuscript. All authors read and approved the final version of the manuscript.

## Conflict of Interest

The authors declare that the research was conducted in the absence of any commercial or financial relationships that could be construed as a potential conflict of interest.

## Footnotes

1 https://portal.gdc.cancer.gov/

2 https://www.gtexportal.org

3 https://www.ncbi.nlm.nih.gov/geo/

4 http://gepia.cancer-pku.cn/

5 https://www.oncomine.org/resource/main.html

6 https://string-db.org/

7 www.kmplot.com

8 www.oncomine.org

9 www.cbioportal.org

10 https://cistrome.shinyapps.io/timer/

11 https://www.r-project.org/

## Supplementary Material

Supplementary Figure 1 The analysis flow chat of this study in finding hub genes of human ovarian cancer.

Supplementary Figure 2 Heatmap of differently expressed genes between human ovarian cancer and normal ovarian tissue.

Supplementary Figure 3 GO and KEGG analysis of differently expressed genes between ovarian cancer and normal ovarian tissue. **(A)** Go enrichment analysis. **(B)** KEGG analysis.

Supplementary Figure 4 Identification of the differentially expressed metabolism-related genes. **(A)** Heatmap of the differentially expressed metabolism-related genes between OC and normal tissue in TCGA. Red, high expression; Green, low expression. **(B)** PPI network of the hub genes based on the STRING database. **(C)** GO enrichment analysis results. **(D)** KEGG pathway enrichment analysis results.

Supplementary Figure 5 Forrest plots of multivariate Cox regression analysis in the training cohort.

Supplementary Figure 6 The expression of seven metabolism-related hub-genes in OC patients. **(A)** The mRNA levels of seven metabolism-related genes in OC (Oncomine). Red, high expression; Blue, low expression. **(B)** OncoPrint summary of genetic alterations of the hub genes.

Supplementary Figure 7 Summary of alteration frequency with high expression **(A)** and low expression **(B)** of seven genes, respectively.

Supplementary Figure 8 Association of immune cells with DGKD **(A)** and IDO1 **(B)** expression in ESTIMATE.

Supplementary Figure 9 Association of immune cells with PNPO expression in ESTIMATE database **(A)** and TIMER database **(B)**.
