# Supplementary material for: Predicting Panel of Metabolism and Immune-Related Genes for the Prognosis of Human Ovarian Cancer
Source: Front Cell Dev Biol. 2021 Jul 12;9:690542. doi: 10.3389/fcell.2021.690542 (PMC8312230; doi:10.3389/fcell.2021.690542)
Supplement: Supplementary file 1 [file Table_1.DOCX]

**Supplementary Table 1**. Sequences of qRT-PCR primers.

| **Name** | **Sense (5’ → 3’)** | **Antisense (5’ → 3’)** |
| --- | --- | --- |
| GFPT2 | ATGTGCGGAATCTTTGCCTAC | ATCGAGAGCCTTGACTTTCCC |
| DGKD | CTTCGAGGGCGAACGCTTTA | TTTTGGTACTGGATTCAGCTACG |
| ACACB | CAAGCCGATCACCAAGAGTAAA | CCCTGAGTTATCAGAGGCTGG |
| ACSM3 | AGGAAGATGCTACGTCATGCC | ATCCCCAGTTTGAAGTCCTGT |
| IDO1 | GCCAGCTTCGAGAAAGAGTTG | ATCCCAGAACTAGACGTGCAA |
| TPMT | TGCGGAAAAGCGGTTGAGAT | AGGAATTTCGGTGATTGGTTCTT |
| PGP | TGACCCGCACTTCAGCTAC | GGGCTTCCCGATGATGTCG |
