# Supplementary figures and images for: Predicting Panel of Metabolism and Immune-Related Genes for the Prognosis of Human Ovarian Cancer
Source: Front Cell Dev Biol. 2021 Jul 12;9:690542. doi: 10.3389/fcell.2021.690542 (PMC8312230; doi:10.3389/fcell.2021.690542)

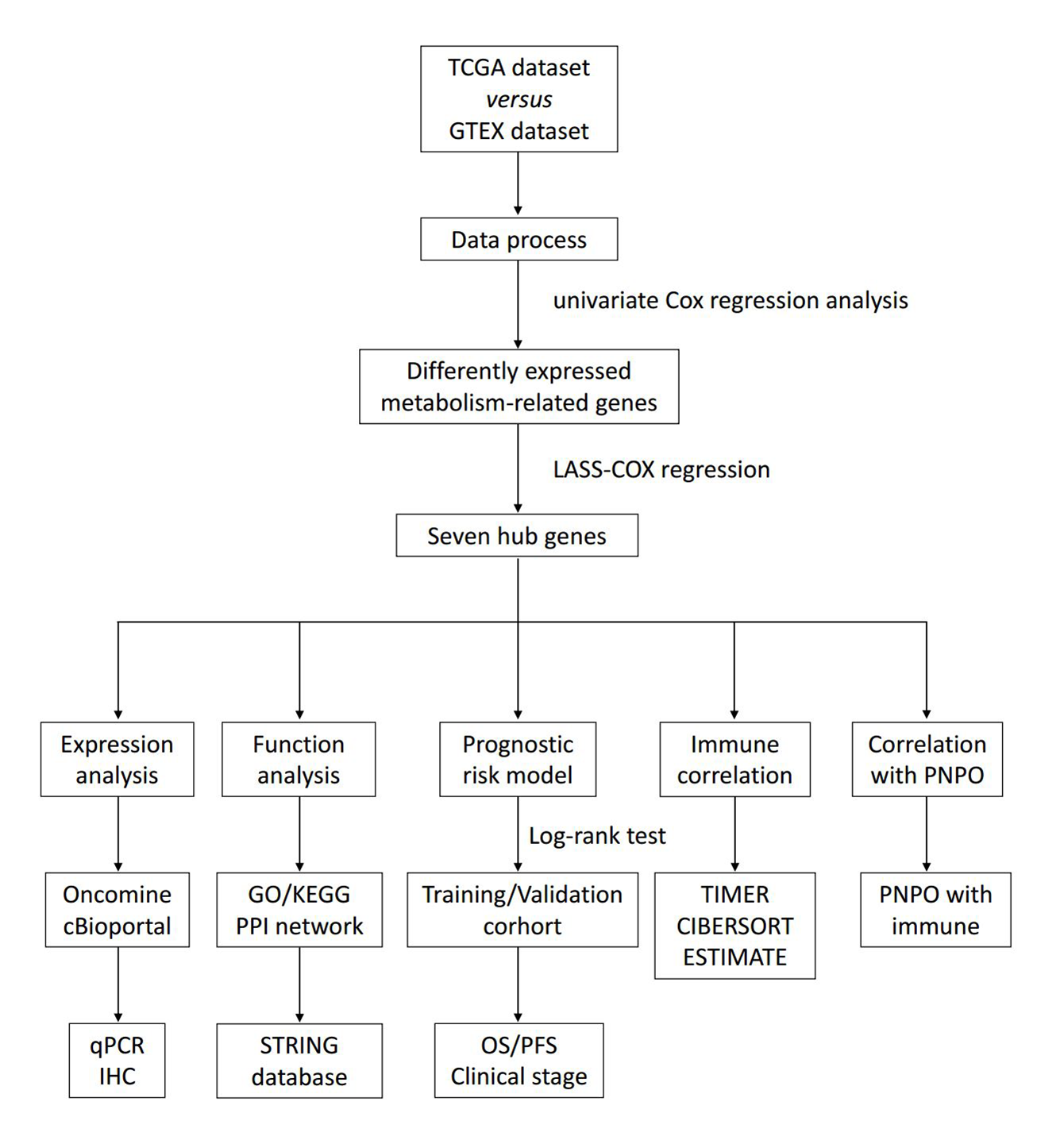

Supplement: Supplementary Figure 1 — The analysis flow chat of this study in finding hub genes of human ovarian cancer. [file Image_1.TIF]

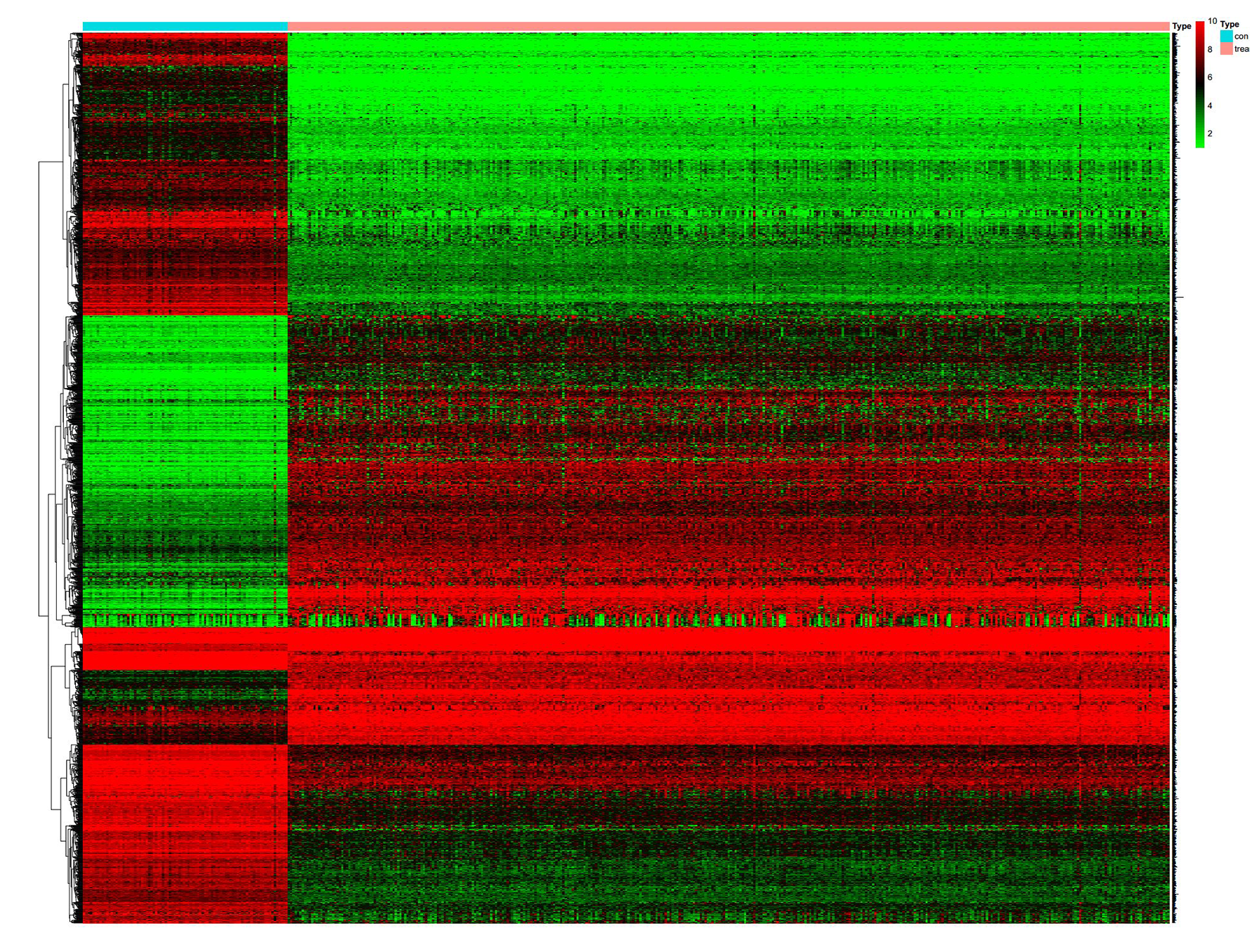

Supplement: Supplementary Figure 2 — Heatmap of differently expressed genes between human ovarian cancer and normal ovarian tissue. [file Image_2.TIF]

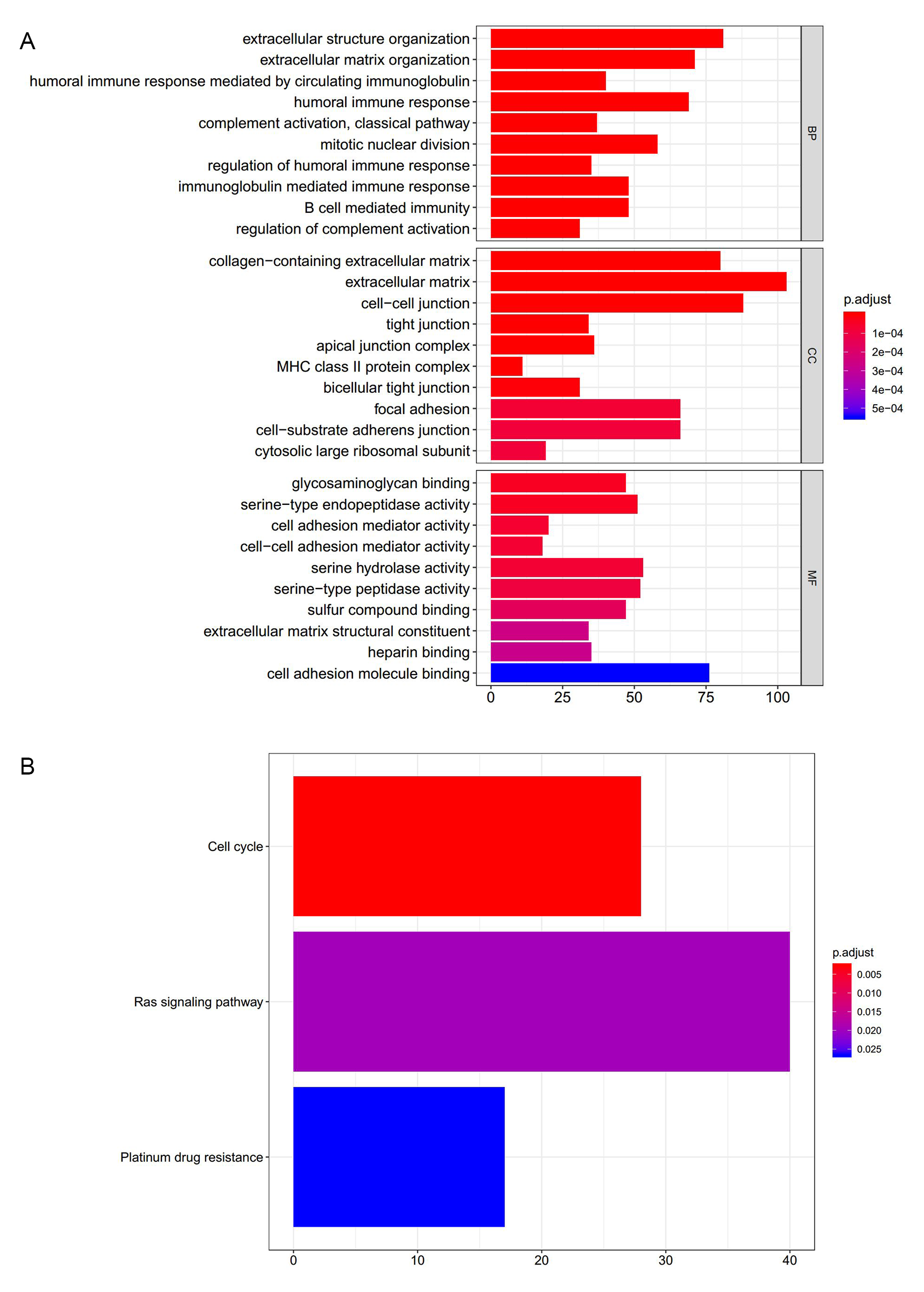

Supplement: Supplementary Figure 3 — GO and KEGG analysis of differently expressed genes between ovarian cancer and normal ovarian tissue. (A) Go enrichment analysis. (B) KEGG analysis. [file Image_3.TIF]

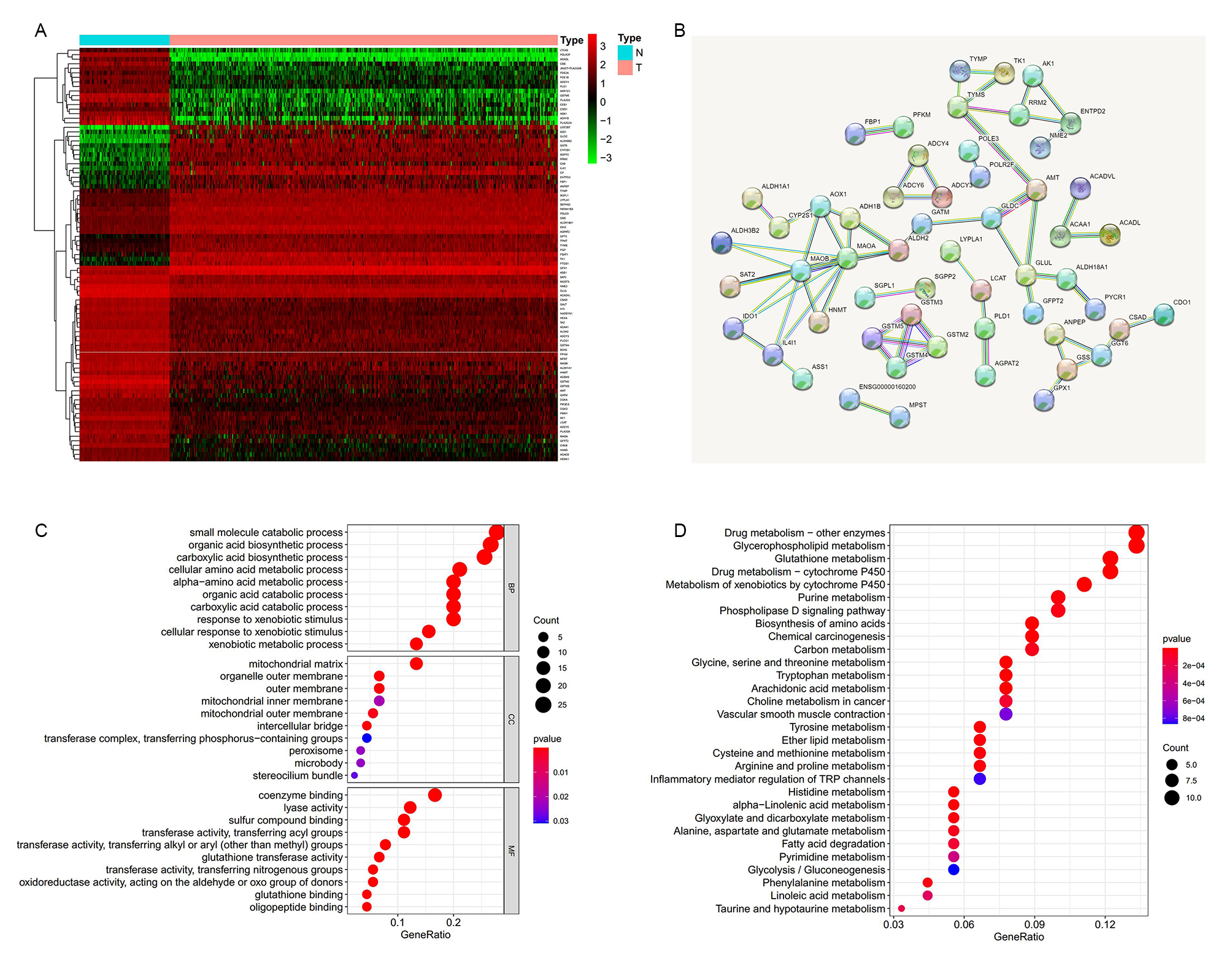

Supplement: Supplementary Figure 4 — Identification of the differentially expressed metabolism-related genes. (A) Heatmap of the differentially expressed metabolism-related genes between OC and normal tissue in TCGA. Red, high expression; Green, low expression. (B) PPI network of the hub genes based on the STRING database. (C) GO enrichment analysis results. (D) KEGG pathway enrichment analysis results. [file Image_4.TIF]

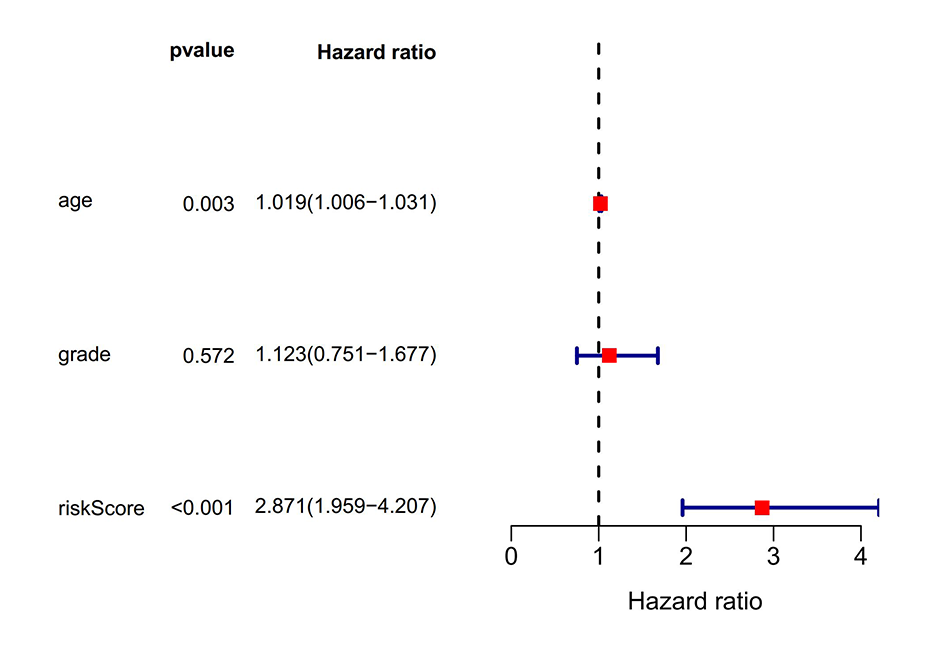

Supplement: Supplementary Figure 5 — Forrest plots of multivariate Cox regression analysis in the training cohort. [file Image_5.TIF]

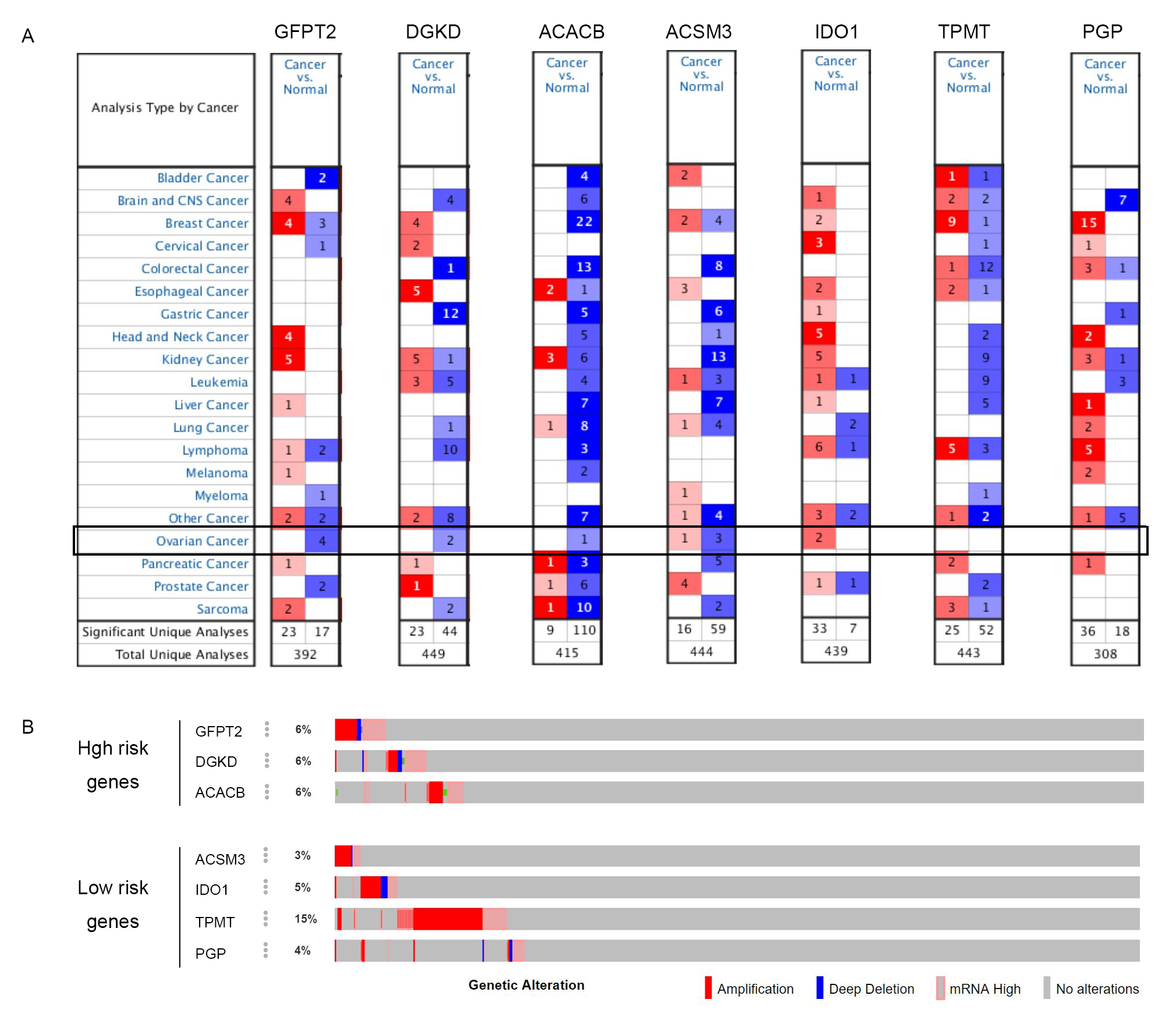

Supplement: Supplementary Figure 6 — The expression of seven metabolism-related hub-genes in OC patients. (A) The mRNA levels of seven metabolism-related genes in OC (Oncomine). Red, high expression; Blue, low expression. (B) OncoPrint summary of genetic alterations of the hub genes. [file Image_6.TIF]

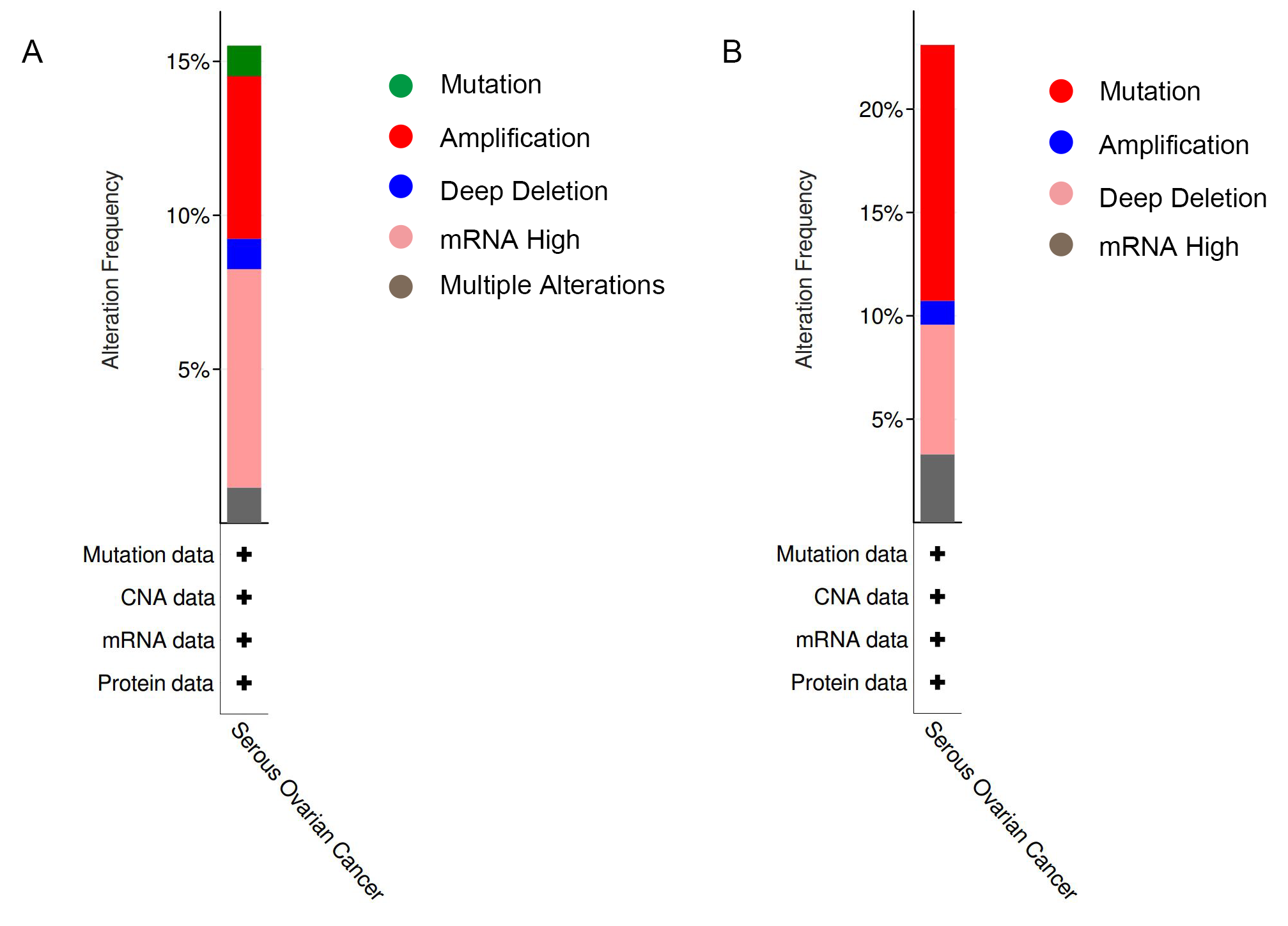

Supplement: Supplementary Figure 7 — Summary of alteration frequency with high expression (A) and low expression (B) of seven genes, respectively. [file Image_7.TIF]

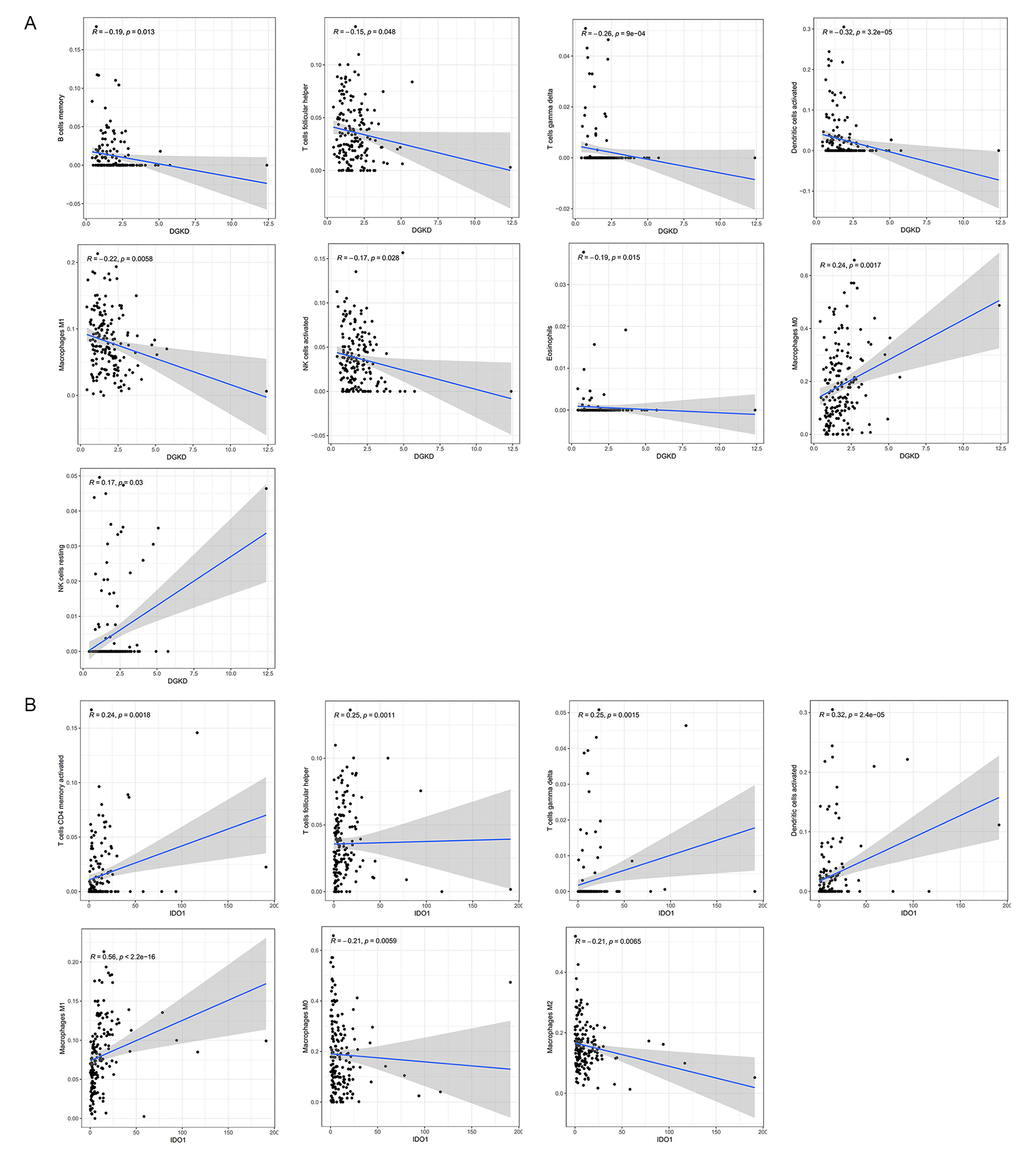

Supplement: Supplementary Figure 8 — Association of immune cells with DGKD (A) and IDO1 (B) expression in ESTIMATE. [file Image_8.TIF]

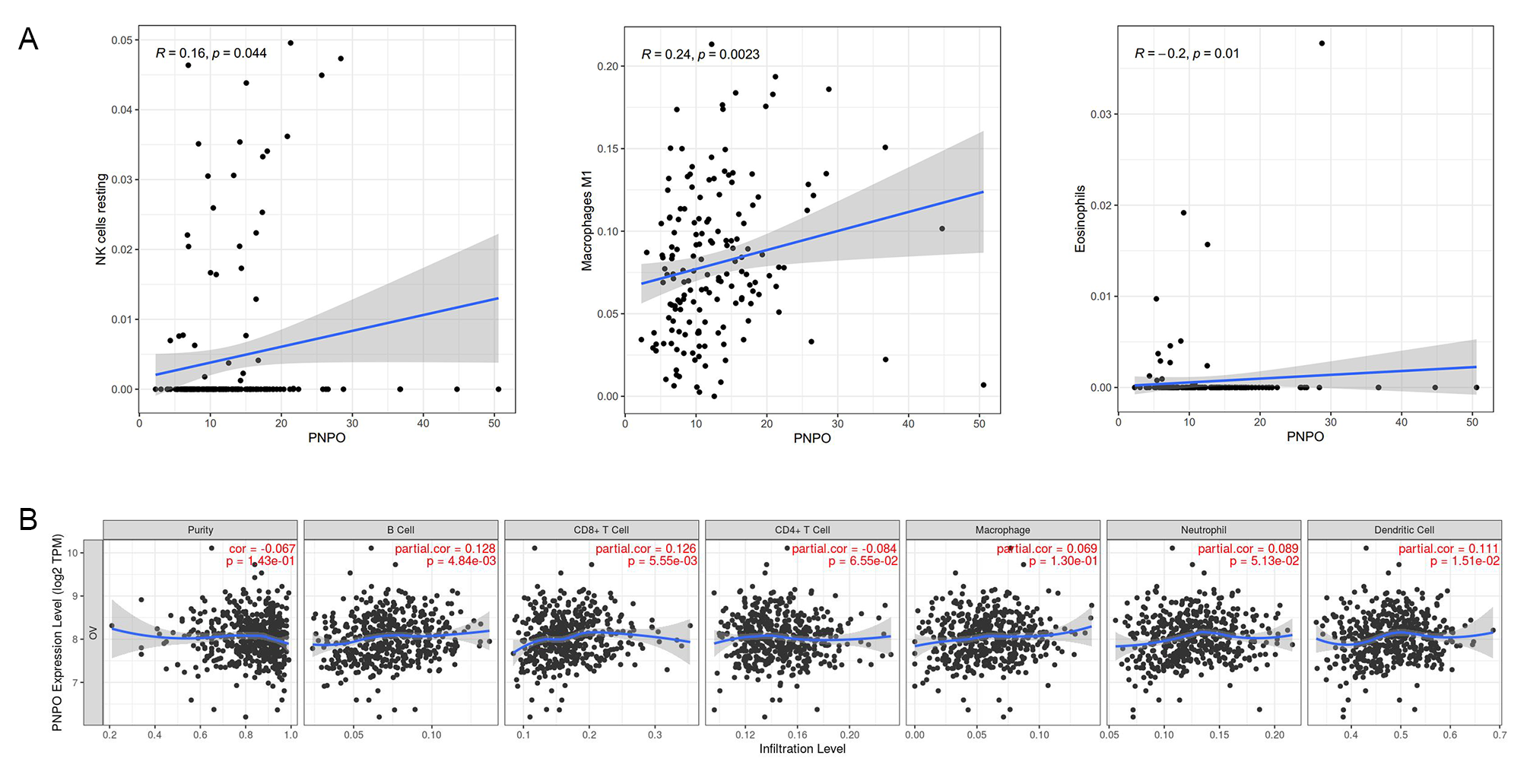

Supplement: Supplementary Figure 9 — Association of immune cells with PNPO expression in ESTIMATE database (A) and TIMER database (B). [file Image_9.TIF]
